# Storms facilitate airborne DNA from leaf fragments outside the main tree pollen season

**DOI:** 10.1007/s10453-024-09826-w

**Published:** 2024-05-22

**Authors:** Mary Hanson, Geoff Petch, Beverley Adams-Groom, Thor-Bjørn Ottosen, Carsten A. Skjøth

**Affiliations:** 1 Edith Cowan University, 270 Joondalup Drive, Joondalup, WA 6027 Australia; 2 School of Science and the Environment, University of Worcester, Henwick Grove, Worcester, WR2 6AJ UK; 3 Danish Technological Institute, Kongsvang Allé 29, 8000 Aarhus C, Denmark; 4 Department of Environmental Science, iCLIMATE, Aarhus University, Frederiksborgvej 399, 4000 Roskilde, Denmark

**Keywords:** Bioaerosols, Abscission, Senescence, Leaves, eDNA

## Abstract

Bioaerosols are useful indicators of plant phenology and can demonstrate the impacts of climate change on both local and regional scales (e.g. pollen monitoring/flowering phenology). Analysing bioaerosols with eDNA approaches are becoming more popular to quantify the diversity of airborne plant environmental DNA (eDNA) and flowering season of plants and trees. Leaf abscission from broadleaved trees and other perennial species can also indicate the status of plant health in response to climate. This happens primarily during autumn in response to seasonal growth conditions and environmental factors, such as changing photoperiod and reduced temperatures. During this period biological material is released in larger quantities to the environment. Here, rural bioaerosol composition during late summer and autumn was captured by MiSEQ sequencing of the rRNA internal transcribed spacer 2 (ITS2) region, a common marker for taxonomic variation. Meteorological parameters were recorded from a proximal weather station. The composition of atmospheric taxa demonstrated that deciduous tree DNA forms part of the bioaerosol community during autumn and, for several common broadleaved tree species, atmospheric DNA abundance correlated to high wind events. This suggests that both flowering and autumn storms cause bioaerosols from deciduous trees that can be detected with eDNA approaches. This is an aspect that must be considered when eDNA methods are used to analyse either pollen or other fragments from trees.

**Supplementary Information:**

The online version contains supplementary material available at 10.1007/s10453-024-09826-w.

## Introduction

Bioaerosols, including pollen, fungi and bacteria, are of well-known significance to anthropogenic activities such as agriculture and urbanisation through their actions as crop pathogens and human allergens (Fröhlich-Nowoisky et al., 2016); however, less is known about patterns of other bioaerosol components, such as leaf fragments, which may occur in the atmosphere as part of natural interactions between plants and environmental factors (Jones & Harrison, 2004). Many bioaerosols can be detected with DNA approaches, with examples covering pollen (e.g. Brennan et al., 2019), bacteria (Innocente et al., 2017) and fungal spores (Hanson et al. 2022a, 2022b). A recent review by Johnson et al. (2023) discussed developments in botanical applications of airborne eDNA detection and highlighted the future potential for advancements in this area, such as monitoring species dispersal, population structures and conservation. Many atmospheric plant studies have focused on wind dispersal and long distance transport of allergenic pollen (e.g. Bayr et al., 2023; Maya-Manzano et al., 2023a, 2023b), often due to its relevance for human health (e.g.Banasiak et al., 2022; Visez et al., 2021); however, it has also been shown that insect pollinated plant species can be detected in atmospheric eDNA (Johnson, et al., 2019).

Abundance of atmospheric pollen is dependent on conditions that affect plant flowering, such as photoperiod and temperature, and this seasonality of plant reproductive processes is captured in pollen calendars (Adams-Groom et al., 2020). Abundance of fungal spores is also often seasonal (Ščevková & Kováč, 2019), however spores are not well represented in bioaerosol monitoring as the number of taxa that can be monitored by microscopy is restricted due to the morphological similarities between many taxa (Galán et al., 2014; Holt & Bennett, 2014). Therefore, understanding the effects of environmental factors and seasonality on bioaerosol communities is currently limited to the most abundant pollen and fungi. Technologically advanced detection and quantification methods, such as automated particle analysers and monitoring devices (Matavulj et al., 2022; Schaefer et al., 2021; Smith et al., 2022) are being developed to improve capture and numeration of selected bioaerosols, while environmental DNA studies (Brennan et al., 2019; Tordoni et al., 2021; Hanson et al., 2022a) are advancing the resolution of bioaerosol taxa identification. Both approaches reduce operator subjectivity as they both rely on numerical approaches that compare the collected bioaerosol with reference libraries. These technologies have not yet been applied for routine monitoring, and international networks collecting data using these advanced methods are in their infancy (Maya-Manzano et al., 2023a, 2023b; Ovaskainen et al., 2020). Data gathered from individual studies using these modern techniques is valuable to answer scientific questions about bioaerosol activity and demonstrate the capabilities for collaborative networks of advanced bioaerosol monitoring. Importantly, it is needed to quantify both possibilities and limitations of these new technologies.

Many studies on bioaerosols focus on specific periods such as transport of bacteria during spring (Abd Aziz et al., 2018), flowering of trees and plants (Adams-Groom et al., 2020) or sporulation and spore release during agricultural production periods (Hanson et al. 2022a, 2022b; Skjøth et al., 2012). A consequence is that the bioaerosol composition outside of flowering periods or agricultural productive periods remains relatively unexplored, although studies such as Aalismail et al. (2021) and Johnson et al. (2021) have demonstrated the potential to advance understanding of plant ecology through monitoring atmospheric plant DNA, with possible relevance of long distance transport and detection of plant fragments, respectively.

New technologies such as next-generation sequencing and automated particle detection are likely to reduce this knowledge gap, bridging the bioaerosol relationships between atmospheric and terrestrial ecosystems. For example, many deciduous tree species abscise their leaves as part of annual nutrient fluxes to aid winter survival and subsequent growth (Patharkar & Walker, 2018). Studying the timing of leaf abscission and associated processes, such as leaf senescence, in association with monitoring environmental parameters can be useful for monitoring the effects of climate change on tree phenology, forest ecosystems and productivity (Gárate-Escamilla et al., 2020; Yang et al., 2021). Remote sensing is often used as a useful proxy for leaf senescence, due to the colour change of leaves as photosynthetic pigments breakdown (Mariën et al., 2019) and can be used to distinguish between coniferous and broadleaved forests (Ottosen et al., 2020); however, it is rarely used to study leaf abscission and physical observations of litter-fall are necessary to complement remote sensing data (as used in Wang et al., (2022) and Gong et al., (2022)). eDNA approaches and sampling bridging atmospheric and terrestrial ecosystems may here provide new opportunities. Here, we tested the relationship between deciduous tree DNA in the atmosphere and environmental parameters during a timeframe associated with leaf abscission.

## Materials and methods

Airborne material was collected from a rural site in Worcestershire (52.2544°, − 2.2537°) using a Burkard multi-vial cyclone sampler. Sampling, sample handling and subsequent processing are detailed in Hanson et al. (2022a), but in brief consisted of daily air sampling using a multi-vial cyclone sampler for eighteen weeks from the end of June to the end of October. Sample tubes were sealed in the field and subsequently handled under aseptic conditions when pooled into weekly samples; a positive control of mixed fungal spores and plant pollen was included along with negative controls for DNA extraction and PCR; DNA was extracted according to Hanson et al. (2022a).

Illumina MiSEQ sequencing was performed (Eurofins Genomics) on the ITS2 region using the primers: forward—5′-GCATCGATGAAGAACGCAGC-3′ and reverse—5′-TCCTCCGCTTATTGATATGC-3′ (Bruns, 1990). Bioinformatic analysis was performed in *R* as detailed in Hanson et al. (2022a) and following the DADA2 ITS workflow l (Callahan et al., 2016, 2017) with taxonomic assignment against the general release UNITE eukaryotic database 29.11.2022 (Abarenkov et al., 2023) followed by phylogenetic analysis using phyloseq and vegan packages (McMurdie & Holmes, 2013; Oksanen et al., 2015). The abundance of plant DNA in the atmosphere was studied by sub-setting the phylum Anthophyta which includes land plants. Diversity in the twenty most abundant genera over the sampling period was assessed using Shannon and Simpson alpha diversity indices.

The environmental variables of rainfall, relative humidity, wind speed, wind direction, temperature, atmospheric pressure were extracted from the Met Office Integrated Data Archive System (MIDAS) for the Pershore Climate Station (52.1001°, − 2.0600°) (Office, 2012). The effect of environmental factors on plant atmospheric community composition at genus taxonomical level was examined by redundancy analysis (RDA). Detrended correspondence analysis (DCA) was applied which showed first axis length of 3.4, suggesting that linear or unimodal ordination could be applied and a linear response was assumed with Hellinger-transformation of genus relative abundances used to reduce weight on low abundance genera and zero counts (Legendre & Gallagher, 2001; Peres-Neto et al., 2006) prior to testing with the RDA function of the vegan package.

Subsequently, Pearson’s correlation was used to examine the relationships between wind speed and air temperature on the abundance of DNA from deciduous trees in the atmosphere. To determine if pollen was a potential source of tree DNA in the atmosphere during the sampling period, a comparison with microscopic counts was subset from the sequence data; *Corylus, Alnus, Salix, Betula, Fraxinus, Quercus* and *Tilia.* The pollen data were counted as part of the UK national pollen monitoring programme using data from University of Worcester (Adams-Groom et al., 2020) located about 6.4 km from the rural trap and hence both of them within the standard pollen dispersal distance of 30 km (Frisk et al., 2022).

## Results

**Fig. 1:**
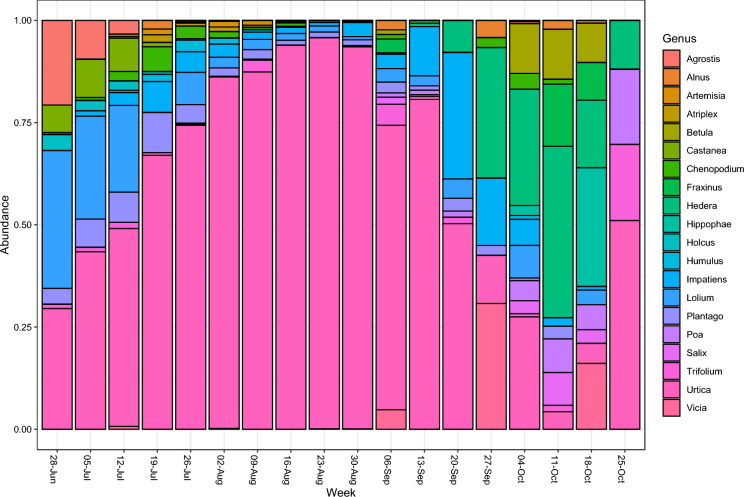
Relative abundance of the top 20 genera in the phylum Anthophyta, from atmospheric DNA samples taken in late summer and autumn (end of June to end of October) at a rural site in Worcestershire, UK, based on rDNA ITS2 profiling

From 3169 taxa within the total of all 18 samples, the subset of Anthophyta comprised 166 taxa. Amongst the most abundant tree genera were oak (*Quercus*), chestnut (*Castanea*), ash (*Fraxinus*) and birch (*Betula*). Other abundant genera included several woody shrubs, flowering perennials and trees and the top 10 are shown in Fig. 1.

Species diversity varied over time, with less diversity observed during the middle of the sampling period which corresponded to the summer month of August. Greater diversity was observed after the start of autumn during late September and through October. This included an increase in abundance of tree DNA within the atmosphere (Table S1).

To study the likelihood of pollen as a source of atmospheric tree DNA, taxa were subset to include only those monitored by the UK pollen network. This allowed comparison of patterns between abundance of atmospheric tree DNA and pollen levels recorded by microscopy. During the sampling period few or no counts were recorded for several genera routinely included in pollen network monitoring, namely *Alnus*, *Betula*, *Corylus* or *Fraxinus,* despite being detected in atmospheric DNA samples. Conversely no *Taxus* was recorded in the atmospheric DNA samples but was recorded by microscopy. *Salix* was absent from both datasets.

**Fig. 2:**
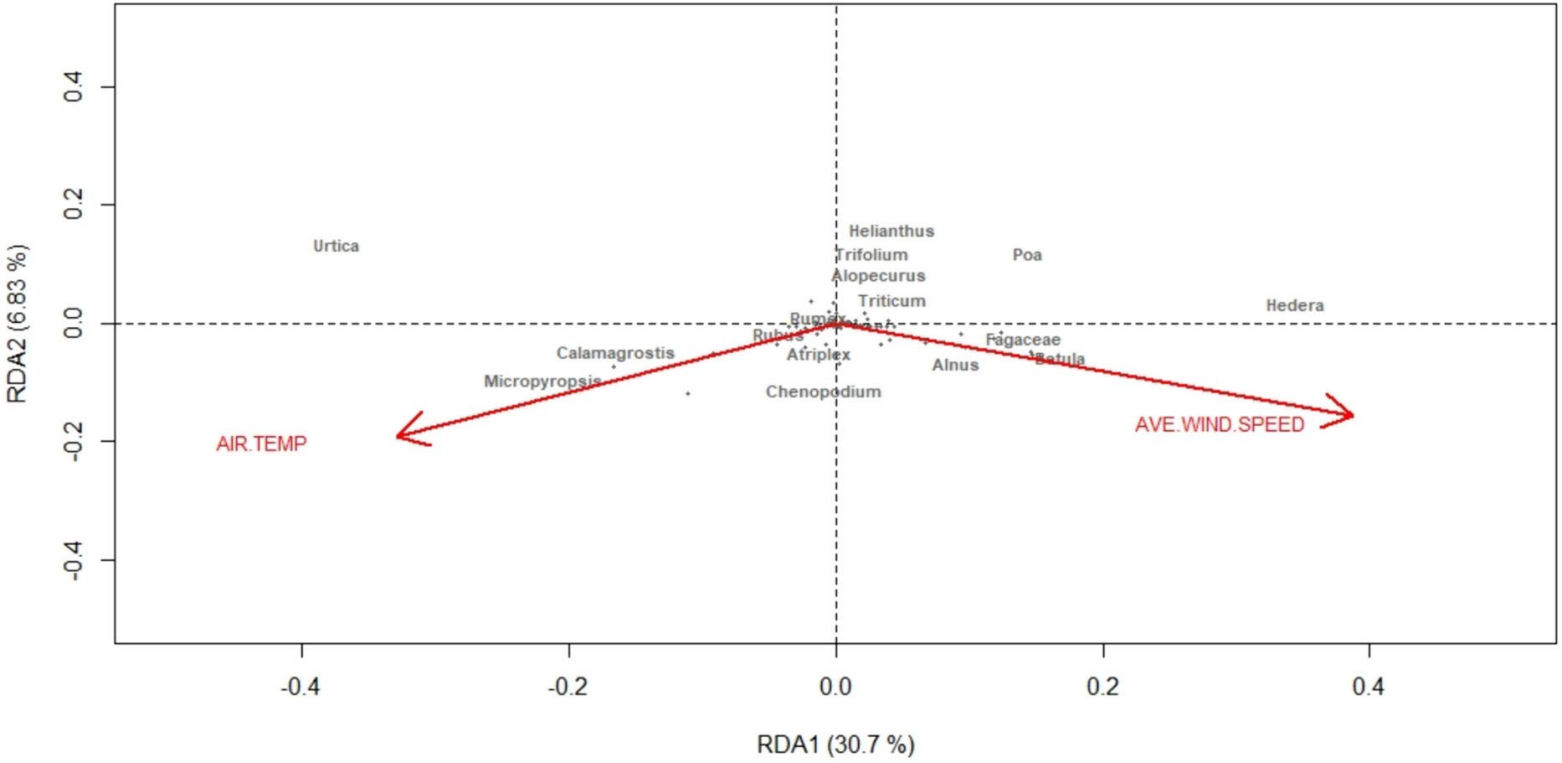
Redundancy analysis (RDA) of environmental factors on atmospheric plant community composition, using Hellinger-transformed genus level data

The environmental variables included in the global RDA model (air temperature, wind speed, relative humidity, wind direction, weekly rainfall and atmospheric pressure) explained 49.9% of the variation in genera abundance (constrained proportion = 0.499). The first RDA axis demonstrated significance of *p* = 0.005 and following forward selection of each variable, wind speed and air temperature were retained as significant in the RDA model (*p* < 0.05) and explained 21.3% of the variation in genera abundance (adjusted *R*^2^ = 0.213) (Fig. 2).

Pearson’s product moment correlation showed that atmospheric DNA from six deciduous trees had significantly positive relationships with wind speed (Table 1). Only one deciduous tree, *Castanea* (Chestnut), showed a significant positive relationship with air temperature and was negatively associated with wind speed, although this was not significant.

**Table 1:** Pearson’s product moment correlation coefficient for relative abundance of atmospheric DNA from deciduous trees against air temperature and average wind speed

Deciduous tree genus	Air temperature	Average wind speed
*Betula*	− 0.28876	0.60585*
*Fraxinus*	− 0.273	0.603089*
*Castanea*	0.478391*	− 0.32921
*Alnus*	− 0.06399	0.50486*
*Salix*	− 0.3136	0.605225*
*Crataegus*	− 0.12593	0.190288
*Fagus*	− 0.21288	0.573749*
*Tilia*	− 0.12994	0.19357
*Quercus*	− 0.27115	0.428563
*Ulmus*	− 0.23899	0.410549
*Acer*	− 0.38372	0.059459
*Prunus*	− 0.23659	0.406882
*Pyrus*	− 0.1702	0.120081
*Citrus*	− 0.23659	0.406882
*Aesculus*	− 0.0695	0.497027*
*Sambucus*	0.382229	− 0.32655

*p value < 0.05

**Fig. 3:**
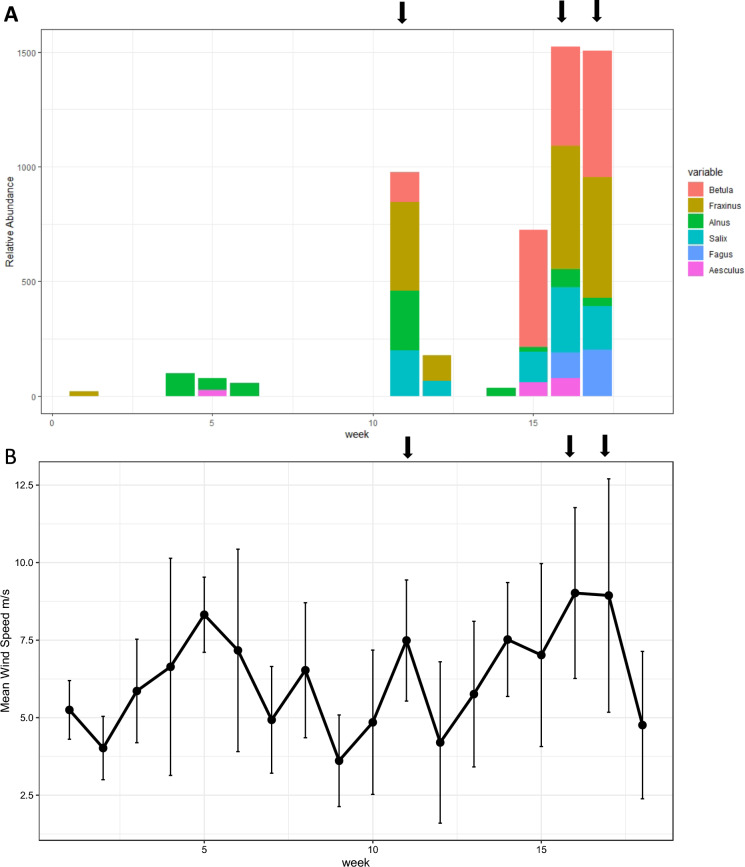
**A** Relative abundance of deciduous tree DNA in the atmosphere over time (weeks) during July–November 2017. Black arrows indicate date of impact of storms on the UK during corresponding timeframe (Storm Aileen 12–13th Sept; Ex-Hurricane Ophelia 16–17th Oct and Storm Brian 21st Oct). **B** Mean wind speed m s^−1^ and weekly standard deviation recorded at Pershore Climate Station during the corresponding timeframe

Peaks of wind speed and atmospheric DNA for deciduous tree species corresponded three times during the sampling period (Fig. 3). A small increase in atmospheric DNA was recorded in weeks 4–6 (19th July–8th Aug), with stronger peaks occurring in weeks 11–12 (6th–19th Sept) and weeks 15–17 (4th–24th Oct) which corresponded with dates of storm impacts in the UK (Met Office https://www.metoffice.gov.uk/weather/warnings-and-advice/uk-storm-centre/uk-storm-season-2017-18).

## Discussion

Bioaerosols are, by definition, airborne biological particles and are considered as mixtures of pollen, fungi, bacteria, algae, viruses and their constituent parts, such as proteins and other fragmented tissues (Gollakota et al., 2021). Angiosperms, the flowering plants, are a major source of bioaerosols due to their reproductive strategy of producing airborne pollen grains, dispersed by wind, for sexual reproduction. Here, the grasses, flowering plants and trees recorded during summer and autumn were largely consistent with typical UK flora, apart from *Citrus* and *Micropyropsis*. The most abundant genus recorded in atmospheric samples was *Urtica* (Nettle). This is not the most abundant by percentage land cover at the sampling site (McInnes et al., 2017) but reaches peak flowering between May and September (Adams-Groom et al., 2020) therefore contributing high pollen load to the overall atmospheric abundance. Grasses (*Poaceae*) show higher percentage cover than *Urtica* but lower abundance in the atmospheric samples, likely due to their earlier flowering peak between May and July (Adams-Groom et al., 2020; McInnes et al., 2017). *Citrus* is not hardy in the UK but often sold at garden centres and nurseries to be grown in pots and taken indoor during the winter period, however, being evergreen it is more probable that a local emission source of agricultural waste includes shredded material from fruit crops, such as citrus. It is unlikely that this was a result of contamination as *Citrus* was not detected in the controls. *Micropyropsis* is a surprising finding, as the only known *Micropyropsis* species, *M. tuberosa* (synonym *Lolium tuberosum*)*¸* is endangered and native to Spain and Morocco. However, ITS sequence data are minimal for the *Lolium* genus, which also contains species common in the UK, such as *Lolium perenne*, and this finding may reflect a limitation of ITS barcodes, as discussed in Mbareche et al. (2020).

Redundancy analysis provides a useful indication of possible associations between environmental factors and species. Here air temperature and wind speed were significant to bioaerosol composition, with several taxa, such as grasses, showing positive associations with air temperature, which is typical of their pollen seasonality (Adams-Groom et al., 2022). Tree species typically flower during spring and summer (Meng et al., 2022) and would be expected to be missed by the late summer–autumn sampling here, however their presence within the top twenty relatively abundant genera, suggests an alternative source of bioaerosol than pollen. This supports the findings of Johnson et al. (2019, 2021) who also observed non wind-pollinated plant species in air samples. Our research goes further by demonstrating both an increased abundance of tree species in the atmosphere during autumn and the association of several deciduous tree genera, whilst wind speed in the RDA can be explained by the prevalence of storms and high wind speeds during this period, in association with the timing of leaf abscission. This process may create small plant fragments, identified as a subset of the bioaerosol (Jones & Harrison, 2004). The annual cycle of leaf growth in deciduous trees cumulates in breakdown of photosynthetic pigments (e.g. chlorophyll, neoxanthin and β-carotene) and macromolecules during leaf senescence followed by abscission of remaining leaf structures. Leaf senescence usually occurs during autumn (Aug–Oct) in Europe (Delpierre et al., 2009; Mariën et al., 2022) in response to environmental factors such as photoperiod or air temperature (Moon et al., 2022) and abscission of the degraded leaves then clears the tree for new leaf growth the following year (Patharkar & Walker, 2018). The breakdown of chlorophyll during senescence is linked to the abscission of remaining leaf structures (Ito et al., 2022), and the process is regulated by hormones such as abscisic acid (Song et al., 2022). Not only are senescence and abscission important for nutrient cycling and plant health, but they are also important processes for woodland productivity and ecosystems through contributions to leaf litter (Wang et al., 2022; Yang et al., 2021). Here we have identified an approach, using eDNA, to quantify remnants from these processes in the atmosphere, although it should be noted that we have not excluded other potential sources of plant eDNA in the atmosphere as little is known about whether plants shed DNA via mechanisms other than leaf abscission.

Monitoring tree health and forest phenology is important for studying the response of forests to changing climate. Presently, leaf abscission is often measured by monitoring leaf fall and studying nutrient fluxes (Wang et al., 2022) while leaf senescence is monitored by proxies, such as (1) remote sensing, where the colour change resulting from photosynthetic pigment degradation is observed from aerial images or (2) monitoring levels of chlorophyll and nitrogen (N) for example, to observe the timing of the start of senescence, when levels will decline (Mariën et al., 2019). Here we find that recording deciduous tree DNA abundance in the atmosphere could provide a novel proxy of the timing and intensity of leaf abscission. Importantly, using eDNA approaches this timing can be done at the species level. A proxy for leaf abscission that can monitor relative abundance of tree DNA abundance may also demonstrate the ability to record patterns over a larger region than physical litter-fall observations.

## Supplementary Information

Below is the link to the electronic supplementary material.

Supplementary file1 (DOCX 14 KB)
