# Supplementary material for: Storms facilitate airborne DNA from leaf fragments outside the main tree pollen season
Source: Aerobiologia (Bologna). 2024 May 22;40(3):415–23. doi: 10.1007/s10453-024-09826-w (PMC11436452; doi:10.1007/s10453-024-09826-w)
Supplement: Supplementary file 1 — Supplementary file1 (DOCX 14 KB) [file 10453_2024_9826_MOESM1_ESM.docx]

|  | Shannon | Simpson |
| --- | --- | --- |
| wk01 | 2.3800089 | 0.8516420 |
| wk02 | 2.0140994 | 0.7702043 |
| wk03 | 2.1923139 | 0.7813723 |
| wk04 | 1.5061084 | 0.5726600 |
| wk05 | 1.2574745 | 0.4642389 |
| wk06 | 0.9755134 | 0.3386871 |
| wk07 | 0.8154954 | 0.2809715 |
| wk08 | 0.6256314 | 0.2003294 |
| wk09 | 0.4642810 | 0.1579859 |
| wk10 | 0.5263060 | 0.1812873 |
| wk11 | 1.8494146 | 0.6231553 |
| wk12 | 1.3207293 | 0.4757339 |
| wk13 | 1.7405208 | 0.7447638 |
| wk14 | 2.2300660 | 0.8655289 |
| wk15 | 2.8066948 | 0.9099316 |
| wk16 | 2.6886041 | 0.9054854 |
| wk17 | 2.6308253 | 0.8897246 |
| wk18 | 1.9949151 | 0.8433459 |

Supplementary table, S1. Shannon and Simpson alpha diversity indices for the top 20 genera with the phylum Anthophyta, over 18 weeks from late June to the end of October, 2017.
